# The Efficacy of Non‐Cultured Epidermal Cell Suspension and Excimer Lamps Combination Therapy in Vitiligo: Results of 18 Months Follow‐Up

**DOI:** 10.1111/jocd.16714

**Published:** 2024-12-16

**Authors:** Tam Hoang Van, Davinder Parsad, Thuong Nguyen Van, Phuong Hoang Thi, Son Nguyen Hong, Hien Do Thi Thu, Tan Nguyen Manh, Hien Le Thanh, Hien Tran Thi Thu, Doanh Le Huu

**Affiliations:** ^1^ Hanoi Medical University Hanoi Vietnam; ^2^ National Hospital of Dermatology and Venereology Hanoi Vietnam; ^3^ Department of Dermatology, Venereology and Leprology Postgraduate Institute of Medical Education and Research Chandigarh India

**Keywords:** Excimer, non‐cultured epidermal cell suspension, non‐segmental vitiligo, segmental vitiligo, vitiligo

## Abstract

**Background:**

Research on evaluating the efficacy of non‐cultured epidermal cell suspension (NCECS) combined with excimer lamps for the treatment of vitiligo is currently unavailable. This research aims to evaluate the efficacy of this combination in treating vitiligo.

**Methods:**

A prospective, controlled study was conducted from November 2021 to January 2024. Patients with stable vitiligo were randomly assigned into groups 1 (NCECS combined with excimer lamps) or 2 (NCECS alone). All patients were followed up 18 months after the procedure. Treatment effectiveness and adverse events were recorded.

**Results:**

Sixty patients were randomly assigned to groups 1 (30 patients) and 2 (30 patients). A total of 33.3% of patients in group 1 achieved 100% repigmentation, significantly higher than the 6.7% in group 2 (*p*‐value = 0.021). Meanwhile, 63.3% of patients in group 1 achieved ≥ 90% repigmentation, higher than the 50% in group 2 but not statistically significant (*p*‐value = 0.435). The mean time to initial repigmentation in group 1 (2.35 ± 0.575 weeks) was significantly shorter than in group 2 (2.72 ± 0.665 weeks) (*p*‐value = 0.003). Both groups demonstrated a similar rate of good color match, but group 1 exhibited a lower incidence of the halo phenomenon. A total of 23.3% of patients in group 1 experienced mild erythema, which spontaneously resolved in a few days.

**Conclusions:**

The combination of NCECS and excimer lamps can substantially stimulate the onset of repigmentation and enhance 100% repigmentation compared to NCECS monotherapy. Excimer lamps may reduce the incidence of the halo phenomenon.

## 1 Introduction

Vitiligo is an acquired pigmentary disorder of unknown origin. The worldwide prevalence of vitiligo was 0.5%–2% [1]. Vitiligo not only affects aesthetics but also substantially impacts the quality of life of patients [2, 3]. Current treatment methods include topical, systemic therapies, phototherapy, and surgical interventions such as skin grafts. However, based on experiences, several patients fail to achieve satisfactory results, particularly in cases of stable vitiligo. Gauthier et al. [4] introduced another method called non‐cultured epidermal cell suspension (NCECS), which demonstrated promising results. After the procedure, patients are recommended to be exposed to sunlight or combined with phototherapy such as ultraviolet A (UVA), ultraviolet B (UVB), and excimer therapy to enhance the effectiveness of this treatment [5, 6]. However, a study revealed that phototherapy may not help enhance the efficacy of NCECS [7]. Literature review revealed no studies on the treatment of vitiligo using NCECS in combination with excimer lamps. Therefore, this research aims to evaluate the efficacy of this combination compared to the treatment with NCECS alone.

## 2 Materials and Methods

### 2.1 Study Design

A prospective, comparative controlled study was conducted on patients with stable vitiligo from November 2021 to January 2024. The patients were divided into two groups based on their day of admission: group 1 comprised patients admitted on even‐numbered days, while group 2 included those admitted on odd‐numbered days. The study procedure received approval from the Ethics Committee. All study interventions were conducted in accordance with established, recognized practices, and all patients (or their guardians) provided informed consent prior to participating in the study.

**FIGURE 1:**
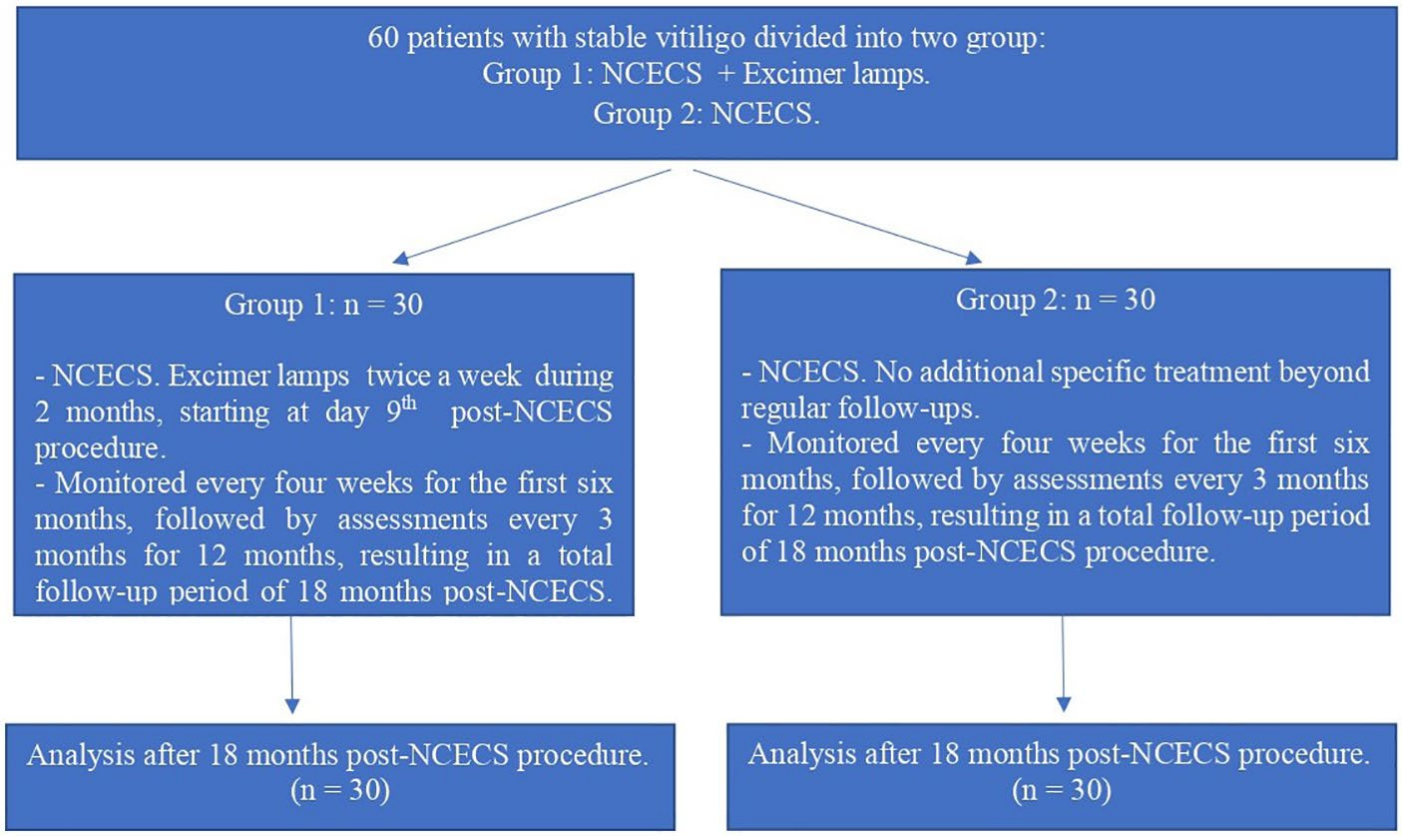
Study flow diagram.

Both patients in groups 1 and 2 were treated with the same intervention of NCECS. After NCECS, only patients in group 1 received phototherapy using excimer lamps. Both groups were monitored every 4 weeks for the first 6 months, followed by assessments every 3 months for 12 months, resulting in a total follow‐up period of 18 months post‐NCECS procedure. The study flow diagram showed in Figure 1. There was no case drop‐out after 18 months follow‐up. The treatment efficacy was assessed based on the repigmentation rate, calculated by comparing the area of lesions before and after treatment. Color match was also evaluated by comparing the color of grafted lesions with the surrounding normal skin color in the clinical evaluations as good color match, hypopigmentation or hyperpigmentation.

### 2.2 Population

Inclusion criteria included patients aged from 18 to 60 year olds diagnosed with segmental or non‐segmental vitiligo, stable for 1 year or more. Stability was defined as having no new lesions and/or no enlargement of existing lesions and/or no occurrence of Koebner's phenomenon for at least 1 year. The area of vitiligo lesions was less than 200 cm^2^. Exclusion criteria included unstable vitiligo, acral vitiligo, mucosal vitiligo, patients with Koebner's phenomenon, and a tendency toward keloid formation.

### 2.3 Study Procedure

All patients were treated with the same intervention of NCECS as follows:

*Step 1*: A thin skin graft was harvested from the upper lateral anterior thigh using a sharp shaving blade. The donor‐to‐recipient‐site ratio of 1:5 was used in this procedure. The donor site was then covered with Bactigras dressing (Smith–Nephew) and a bandage.

*Step 2*: The graft was immersed in a 4% trypsin solution containing ethylene diamine tetraacetic acid and incubated in a CO_2_ incubator at 37°C for 60 min. The skin sample was then transferred to a bacterial culture plate and washed twice with phosphate‐buffered saline (PBS). The dermis was discarded, and the epidermis was dissociated into small pieces. The resulting cell suspension was placed in a falcon tube and centrifuged at 1500 rotations per minute for 6 min. After centrifugation, the precipitate was collected and then suspended in a PBS solution until reaching a volume of 0.1–0.5 mL. This suspension was then placed in a 1 mL syringe with an 18G needle.

*Step 3*: The recipient site was prepared by dermabrasion using CO_2_ laser Pentagon (spot size of 70–120 μm) (Daeju Meditech Engineering, Korea), fractional mode, energy of 200 mJ, density of 1 mm and 1 pass. The epidermis was then ablated using a moistened dressing. The dermabrasion extended 2 mm beyond the margin of vitiligo lesions to minimize the halo phenomenon.

*Step 4*: The suspension containing epidermal cells was then transplanted into the prepared recipient sites. A dry dressing of Neuskin‐F (Eucare Pharmaceuticals) was applied to the entire lesions, followed by Bactigras dressing (Smith–Nephew) and sterilizing dressing, and was then secured with Tegaderm HP (3M) bandage. The dressings were removed from the donor and recipient sites after 1 week.

*Step 5*: Patients in group 1 received phototherapy using excimer lamps (TheraBeam UV308, Ushio, Japan) twice a week with the initial dose of 150 mJ/cm^2^, which was then increased by 50 mJ/cm^2^ each session until reaching the minimal erythema dose. The dose was adjusted based on the erythema reaction. The phototherapy regimen lasted for 8 weeks. Patients in group 2 received no additional specific treatment beyond regular follow‐ups.

### 2.4 Statistical Analysis

Data were collected, reviewed, coded, and analyzed using the Statistical Package for Social Science (SPSS) version 25. Data were summarized using descriptive statistics and frequency tables. The Chi‐squared, Fisher exact, and Mann–Whitney *U* tests were used for comparison as appropriate. A *p*‐value of < 0.05 was considered statistically significant.

## 3 Results

Sixty patients with stable vitiligo were included in this study and divided into two groups. Most of these patients had segmental vitiligo on the face. Table 1 summarizes the characteristics of the two groups.

**TABLE 1:** Clinical and demographic characteristics of two groups.

Data	Group 1 (*n* = 30)	Group 2 (*n* = 30)	*p*
Age	29.23 ± 10.40	28.67 ± 6.87	0.760^a^
Gender (female/male)	16/14	19/11	0.432^b^
Duration of vitiligo (years)	9.77 ± 7.33	9.67 ± 7.57	0.944^a^
Duration of stability (years)	6.50 ± 6.55	7.26 ± 7.23	0.578^a^
Types of vitiligo (segmental/other)	24/6	27/3	0.731^c^
Sites of lesions (face/other)	28/2	29/1	0.554^c^
Area of lesions (cm^2^)	26.9 ± 15.92	30.1 ± 15.91	0.361^a^

^a^ Mann–Whitney *U* test.

^b^ Chi‐square test.

^c^ Fisher exact test.

**FIGURE 2:**
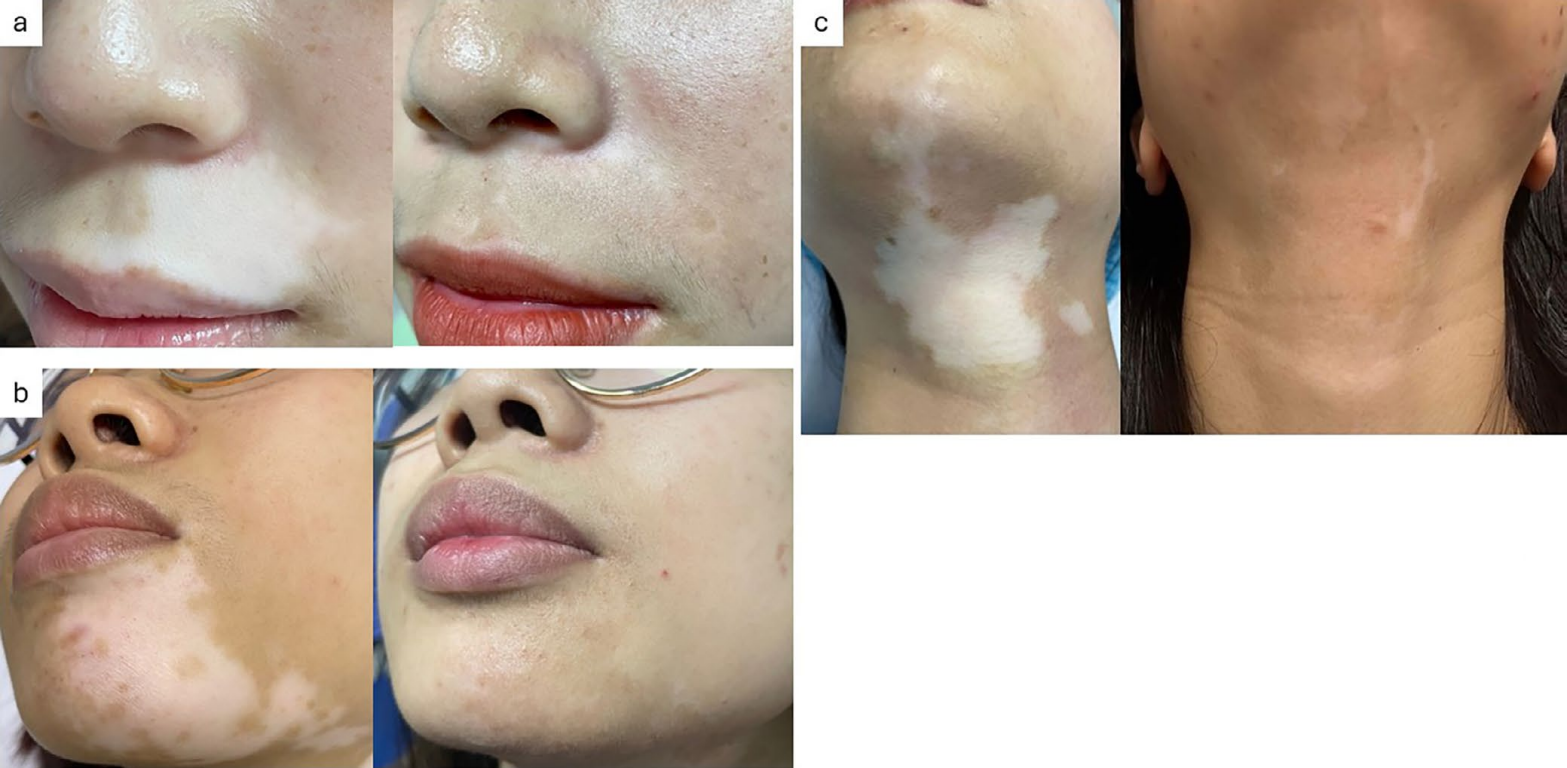
Patient before and at 18 months after treatment. (a, b) Segmental vitiligo in group 1: 100% repigmentation and good skin color match. (c) Segmental vitiligo in group 2: > 90% repigmentation with halo phenomenon.

The treatment response at 18 months after NCECS was presented in Table 2. In group 1, the percentage of patients achieving ≥ 50% repigmentation was 90%, which was equivalent to group 2. Similarly, the percentage of patients achieving ≥ 75% repigmentation in group 1 (73%) was comparable to group 2 (70%). More patients in group 1 achieved ≥ 90% repigmentation (63.3%) compared to group 2 (50%); however, this difference was not statistically significant (*p* = 0.435). Notably, 10 out of 30 patients in group 1 achieved 100% repigmentation (Figure 2a,b), representing 33.3%, which was significantly higher than the 6.7% in group 2 (*p* = 0.021).

**TABLE 2:** Repigmentation at 18 months.

Repigmentation	Group 1 (*n* = 30)	Group 2 (*n* = 30)	*p*
< 50% repigmentation	3 (10%)	3 (10%)	1.000^b^
≥ 50% repigmentation	27 (90%)	27 (90%)	1.000^a^
≥ 75% repigmentation	23 (73.3%)	23 (70%)	1.000^a^
≥ 90% repigmentation	19 (63.3%)	15 (50%)	0.435^a^
100% repigmentation	10 (33.3%)	2 (6.7%)	0.021^b^

^a^ Chi‐squared test.

^b^ Fisher exact test.

Table 3 shows the mean time of onset of repigmentation in both groups. In group 1, the mean time of onset observed was 2.35 ± 0.575 weeks, which was shorter than the 2.72 ± 0.665 weeks observed in group 2 (*p* = 0.003). A total of 66.7% of patients in group 1 experienced repigmentation within 2 weeks, compared to only 36.7% in group 2.

**TABLE 3:** Mean time of onset of repigmentation.

Group	Time of onset of repigmentation (weeks)		
	Mean	Median	*p*
Group 1	2.35 ± 0.575	2	0.003^a^
Group 2	2.72 ± 0.665	3	

^a^ Mann Whitney *U* test.

Regarding the color match, the percentage of good color match exhibited insignificant differences in both groups: 76.7% and 73.3% in groups 1 and 2, respectively. The halo phenomenon was statistically less frequent in group 1 (20%) compared to group 2 (50%) (*p* = 0.029; Table 4 and Figure 2c).

**TABLE 4:** Color match.

Type of color match	Group 1	Group 2	*p*
Good match	23 (76.7%)	22 (73.3%)	1.000^a^
Hypopigmentation	1 (3.3%)	1 (3.3%)	1.000^b^
Hyperpigmentation	6 (20%)	7 (23.4%)	1.000^a^
Halo phenomenon	6 (20%)	15 (50%)	0.029^a^

^a^ Chi‐square test.

^b^ Fisher exact test.

None of the 60 patients in this study experienced side effects such as scarring or infection. However, in group 1, 7 patients (23.3%) reported mild erythema, which spontaneously resolved within a few days without any further complications.

At 18 months, recurrence at the graft sites was observed in two patients in group 1: one patient with segmental vitiligo had a small depigmented lesion, while another patient with non‐segmental vitiligo experienced recurrence at the graft sites as well as on other lesions. In group 2, one patient with segmental vitiligo experienced small lesion recurrence.

## 4 Discussion

Stable vitiligo is often resistant to conventional treatments. Surgical interventions, including NCECS, play a crucial role in managing this condition. These therapies aim to induce repigmentation and minimize the contrast between normal skin and vitiligo lesions. While NCECS has demonstrated promising results in the literature, some studies report a relatively low repigmentation rate and a high risk of the halo phenomenon [5, 8]. The combination of phototherapy modalities, such as UVB, UVA, or excimer therapy, can enhance repigmentation and reduce the halo phenomenon [9, 10]. A comprehensive approach to the management of stable vitiligo lies in the combination of surgical intervention with phototherapy.

The current study showed that patients in group 1 had initial repigmentation earlier than those in group 2 (2.35 ± 0.575 weeks vs. 2.72 ± 0.665 weeks; *p* = 0.003). Therefore, mature melanocytes increase melanin production after transplantation onto vitiligo lesions and stimulation by excimer lamps, resulting in repigmentation. This stimulation can enhance the release of basic fibroblast growth factor and endothelin‐1 (ET‐1) from keratinocytes. Notably, the simulation also promotes the migration of melanocytes through the stimulated expression of phosphorylated focal adhesion kinase (p125FAK) on melanocytes and matrix metalloproteinase‐2 activity from melanocytes [11]. Otherwise, NB‐UVB and excimer induce the apoptosis of T‐lymphocytes, thereby preventing the destruction of newly transplanted melanocytes through immune actions [12]. Most studies show that pigmentation recovery time with NCECS alone is generally within the first month, mainly at week 4 [12, 13]. The onset of repigmentation occurred within only 2 weeks among 66.7% of patients in group 1, whereas that for group 2 took approximately 3 weeks. Compared to other studies, the onset is earlier because excimer light was used on day 2 after dressing removal. Additionally, the donor‐to‐recipient‐site ratio plays a role in initial repigmentation. A high ratio tends to result in early repigmentation. In the study of Tawfik, 88% of lesions began repigmentation within 2 weeks in the group with a ratio of 1:3, which is notably higher compared to only 7.7% in the group with a ratio of 1:10 [14]. The 1:5 ratio used in this study is another factor that reduced the initial repigmentation time.

The repigmentation rates of ≥ 50% and ≥ 75% were comparable between both groups. In group 1, the repigmentation rate of ≥ 90% was 63.3% higher than the 50% observed in group 2, but this difference was not statistically significant (*p* = 0.435), possibly due to the small sample size. However, the 100% repigmentation rate was significantly higher in group 1 compared to group 2 (33.3% vs. 6.7%, *p* = 0.021). These results indicate a superior response in the group that received the combination of NCECS with excimer. This finding may be explained by the role of NB‐UVB or excimer in stimulating melanocyte activities and preventing their apoptosis under the influence of T‐lymphocyte immune responses [12]. These results align with the study conducted by Ebadi et al. [5], who demonstrated that the combination of NCECS and excimer laser was more promising compared to NCECS or excimer laser therapy alone. Their study included 39 vitiligo lesions divided into the following four groups: excimer laser, NCECS, NCECS combined with excimer laser, and a control group. In their study, Ebadi et al. [5] utilized excimer laser therapy for a total of 24 sessions, with a frequency of two to three sessions per week, starting from week 2 after the intervention of NCECS. The combination group in the study of Ebadi exhibited a mean repigmentation of 41.9%, which was significantly higher than the 15.9% observed in the group receiving NCECS alone (*p* < 0.001). However, the repigmentation rate was lower compared to the current study. This result can be explained as follows: firstly, most of the patients in their study had non‐segmental vitiligo, whereas 80% of the patients had segmental vitiligo. Secondly, their study included lesions on the trunk, whereas the current study had more lesions on the face, which are often more responsive to treatment with NCECS. Finally, the use of excimer laser and lamps also made a difference in the results between the two studies. Compared to other studies investigating the combination of NCECS and NB‐UVB, 63.3% of patients in group 1 achieved repigmentation of 90%–100% at 18 months, similar to the findings reported by Zhang et al. [12] The author also emphasized that using NB‐UVB not only after but before the procedure can enhance the effectiveness of the treatment.

Studies in the literature that specifically combined NCECS with excimer light therapy have not been found. Only one study, conducted by Yadav et al. [15], explored the efficacy of excimer light therapy on patients who were partially responsive to different surgical interventions after at least 6 months. These surgical interventions included NCECS (11 cases), NCECS with extracted hair follicle outer root sheath cell suspension (6 cases), suction blister graft (3 cases), and punch graft (2 cases). The study reported a response rate of 27.6% ± 29.2%. However, this study notably lacked a control group, had a small sample size, and included other therapies such as topical tacrolimus and fluocinolone, increasing the difficulty in evaluating the efficacy of excimer light.

Concerns regarding the risk of hyperpigmentation in transplanted lesions upon exposure to ultraviolet light have emerged. However, based on the observations, at 18 months, the color match was 76.7% and 73.3% in groups 1 and 2, respectively, with no significant difference between the two groups. In this study, excimer light therapy was administered for only 8 weeks, after which the color gradually normalized to match that of normal skin. The rate of color match in this study was lower than that in the study of Tawfik, which was 86% [14]. The variability in color match response across studies can be attributed to multiple factors, such as the donor‐to‐recipient ratio, recipient site preparation technique, and post‐transplantation phototherapy. Among these factors, the recipient site preparation technique plays a crucial role. Excessive dermabrasion depth can result in persistent hyperpigmentation in recipient sites. A fractional CO_2_ laser was used for dermabrasion with an energy of 200 mJ, a density of 1 mm and 1 pass. The choice of such a high energy level was influenced by the findings of Oh et al. [16], who used fractional CO_2_ laser (Ultra pulse, Lumenis, spot size of 350 μm). Additionally, Lommerts et al. [17] discovered that a low energy level did not induce the repigmentation effect when using fractional CO_2_ laser (Ultrapulse, Lumenis) set at 7.5 mJ/microbeam, 20% density, and a spot size of 120 μm. Post‐laser hyperpigmentation may be influenced by the density and the energy of the laser [18]. High‐density laser spots can effectively remove the epidermis but also carry a high risk of post‐laser hyperpigmentation. Compared to CO_2_ laser machines with the same spot size, a density of 1 mm was employed, which is lower than that utilized in other studies while using higher energy levels [17, 19]. However, even with this density, 20% and 23.4% of patients in groups 1 and 2, respectively, experienced hyperpigmentation compared to normal skin. This phenomenon was attributed to the possibility of post‐inflammatory hyperpigmentation due to high energy levels of 200 mJ. By contrast, Silpa‐Archa et al. [19] reported a higher incidence of hyperpigmentation (39%) following fractional CO_2_ laser dermabrasion compared to the findings. They used the same energy level of 200 mJ but with a higher density (82%), which likely contributed to the elevated hyperpigmentation rate.

The halo phenomenon, characterized by a white ring around a grafted lesion, often occurs after surgical treatments for vitiligo [8]. Several authors recommend extending dermabrasion 2 mm beyond the lesion margins to prevent this occurrence. Moreover, ultraviolet therapy after surgery has been demonstrated to decrease its incidence [9]. In this study, the occurrence of the halo phenomenon was significantly lower in group 1 compared to group 2, with rates of 20% (6 out of 30 patients) and 50% (15 out of 30 patients), respectively (*p* = 0.029). This effect was more evident in patients with hyperpigmentation. The lower incidence in group 1 may be attributed to the suppression of T‐lymphocytes in the lesion margins by excimer light. The findings are consistent with a study by Al‐Mutairi et al. [10], who employed excimer laser therapy post‐surgically following thin skin grafting for vitiligo treatment. In comparison to the study conducted by Munish et al. [20], the rate of halo phenomenon in group 1 was lower (20% compared to 25.5%). The author provided PUVA only to patients experiencing delayed repigmentation or hypopigmentation, which differs from the early use of excimer light after 2 day of dressing removal. This approach could potentially contribute to the observed reduction in the halo phenomenon. Dev et al. [8] observed the halo phenomenon in 42.3% and 30.8% of patients in the manual dermabrasion and electrofulguration dermabrasion groups, respectively. In this study, the authors did not possibly use any phototherapy, thereby leading to a higher incidence of halo ring compared to group 1. However, in comparison to group 2 (with 50% incidence), the lower rate in this study may be related to the variation of dermabrasion techniques. By contrast, Mulekar et al. [21] observed a lower incidence of halo ring, affecting only 8.16% of patients. They did not report the combination with any phototherapy but used a dermabrader fitted for recipient sites, while this study employed fractional CO_2_ laser. The difference in results observed between the two studies could be attributed to this distinction in preparation recipient sites.

## 5 Conclusion

The early combination of excimer light therapy after dressing removal following the NCECS procedure has the potential to enhance the onset of repigmentation and the efficacy of 100% repigmentation compared to NCECS alone. Additionally, excimer light therapy may help reduce the incidence of the halo phenomenon in grafted lesions.

## Author Contributions

T.H.V., D.L.H., and D.P. designed the research study. T.H.V., T.N.V., P.H.T., S.N.H., H.D.T.T., T.N.M., H.L.T., and H.T.T.T. performed the research. T.H.V., T.N.V., P.H.T., S.N.H., and H.D.T.T. contributed to data collection. T.H.V., T.N.M., H.L.T., and H.T.T.T. analyzed the data. T.H.V. and D.L.H. wrote the paper. T.H.V., D.L.H., and D.P. carried out critical revision. All authors have read and agreed to the published version of the manuscript.

## Ethics Statement

The study procedure received approval from the Ethics Committee of Hanoi Medical University (No: 487/GCN‐HDDDNCYSH‐DHYHN, dated 18/11/2021). All study interventions were conducted in accordance with established, recognized practices, and all patients (or their guardians) provided informed consent prior to participating in the study.

## Conflicts of Interest

The authors declare no conflicts of interest.

## Data Availability

The data that support the findings of this study are available from the corresponding author upon reasonable request.
