# Correction to “The Efficacy of Non‐Cultured Epidermal Cell Suspension and Excimer Lamps Combination Therapy in Vitiligo: Results of 18 Months Follow‐Up”

**DOI:** 10.1111/jocd.70179

**Published:** 2025-05-09

T. Hoang Van, D. Parsad, T. N. Van, et al., “The Efficacy of Non‐Cultured Epidermal Cell Suspension and Excimer Lamps Combination Therapy in Vitiligo: Results of 18 Months Follow‐Up,” *Journal of Cosmetic Dermatology* 24 (2025): e16714, https://doi.org/10.1111/jocd.16714.

1. **Error in calculating the percentage**
  – Original text/data: 23 (73.3%)–23 (70%).
  – Corrected text/data: 23 (76.7%)–23 (76.7%).
  – Location: Result (text), Table 2.
  – Reason for correction: Miscalculation in percentage.
2. **Error in *p* value = 0.554**
  – Original text/data: *p* value = 0.554.
  – Corrected text/data: *p* value = 1.000.
  – Location: Table 1.
  – Reason for correction: Typographical.

These corrections do not alter the overall conclusions of the study. However, they were made to ensure transparency and accuracy in the scientific record.

We sincerely apologize for these oversights.

